# Conceptual framework for designing video games for children with type 1 diabetes^*^

**DOI:** 10.1590/1518-8345.2764.3090

**Published:** 2019-03-18

**Authors:** Valéria de Cássia Sparapani, Sidney Fels, Noreen Kamal, Lucila Castanheira Nascimento

**Affiliations:** 1 Universidade Federal de Santa Catarina, Departamento de Enfermagem, Florianópolis, SC, Brazil.; 2 University if British Columbia, Department of Electrical and Computer Engineering, Vancouver, BC, Canada.; 3 University of Calgary, Department of Clinical Neurosciences, Calgary, AB, Canada.; 4 Universidade de São Paulo, Escola de Enfermagem de Ribeirão Preto, PAHO/WHO Collaborating Centre for Nursing Research Development, Ribeirão Preto, SP, Brazil.

**Keywords:** Diabetes Mellitus, Type 1, Video Games, Pediatric Nursing, Children, Health Behavior, Research, Diabetes Mellitus Tipo 1, Jogos de Vídeo, Enfermagem Pediátrica, Criança, Comportamentos Saudáveis, Pesquisa, Diabetes Mellitus Tipo 1, Juegos de Video, Enfermería Pediátrica, Niño, Conductas Saludables, Investigación

## Abstract

**Objective:**

to present a theoretically based conceptual framework for designing video games for children with type 1 diabetes mellitus.

**Methods:**

this was a methodological study that developed a conceptual framework with nine steps in view of health behavior change theories and the user-centered design approach as theoretical and methodological frameworks, respectively. Twenty-one children, aged 7 to 12 years, participated by expressing their needs and preferences related to diabetes and video games. Data were analysed following content analysis guidelines. Then, a choice of appropriate health behavioral change theories and their determinants that should be capable of influencing children’s behaviors and preferences.

**Results:**

the conceptual framework proposes a video game that consists of six phases, each addressing one stage of behavioral change and specific determinants, aligned with the needs and preferences identified by the participating children. This study shows the applicability of this framework in view of each proposed phase presenting examples and the children’s ideas.

**Conclusion:**

the results of this study contribute to advance the discussion on how behavioral theories and their determinants should be related to the design of creative and funny video games considering the profile of the target population as well as its needs and preferences.

## RESUMO

**Objetivo:**

apresentar uma estrutura teórica conceitual para o desenvolvimento de videogames para crianças com diabetes mellitus tipo 1.

**Métodos:**

este estudo metodológico desenvolveu uma estrutura conceitual com nove etapas, baseada em teorias de mudança de comportamentos em saúde e na abordagem do desenho centrado no usuário como referencial teórico e metodológico, respectivamente. Vinte e uma crianças entre 7 a 12 anos participaram do estudo, expressando suas necessidades e preferências relacionadas ao diabetes e videogames. Os dados foram analisados de acordo com as diretrizes de análise de conteúdo. Em seguida, foram selecionadas teorias de mudança de comportamentos em saúde e seus determinantes, que pudessem ser capazes de influenciar os comportamentos e preferências das crianças.

**Resultados:**

a estrutura conceitual propõe um videogame composto por seis fases, cada uma tratando de um estágio de mudança de comportamento e com determinantes específicos, alinhados às necessidades e preferências identificadas. O estudo mostra a aplicabilidade da estrutura desenvolvida, considerando cada fase proposta, apresentando exemplos e ideias das crianças.

**Conclusão:**

os resultados da pesquisa contribuem para o avanço nas discussões de como as teorias comportamentais e seus determinantes devem estar relacionados ao desenho de videogames criativos e divertidos, considerando o perfil da população alvo, assim como suas necessidades e preferências.

## RESUMEN

**Objetivo:**

presentar una estructura teórica conceptual para el desarrollo de videojuegos para niños con diabetes mellitus tipo 1.

**Métodos:**

este estudio metodológico desarrolló una estructura conceptual con nueve etapas, basada en teorías de cambio de comportamientos en salud y en el enfoque del diseño centrado en el usuario como referencial teórico y metodológico, respectivamente. Veintiún niños entre 7 y 12 años participaron del estudio, expresando sus necesidades y preferencias relacionadas a la diabetes y a los videojuegos. Los datos fueron analizados de acuerdo con las directrices del análisis de contenido. Enseguida, fueron seleccionadas las teorías de cambio de comportamientos en salud y sus determinantes, que pudieran ser capaces de influenciar los comportamientos y preferencias de los niños.

**Resultados:**

la estructura conceptual propone un videojuego compuesto por seis fases, cada una tratando de una etapa de cambio de comportamiento y con determinantes especificos, alineados a las necesidades y preferencias identificadas. El estudio muestra la aplicabilidad de la estructura desarrollada, considerando cada fase propuesta, presentando ejemplos e ideas de los niños.

**Conclusión:**

los resultados de la investigación contribuyen para el avance en las discusiones de como las teorías comportamentales y sus determinantes deben estar relacionados al diseño de videojuegos creativos y divertidos, considerando el perfil de la población objetivo, así como sus necesidades y preferencias.

## Introduction

Type 1 diabetes mellitus (T1DM) is a chronic disease that affects mainly children and adolescents < 18 years old^1^, it has become an international public health with increasing worldwide incidence^2^ ^-^ ^3^. The success of T1DM treatments requires multidisciplinary teams involving and enabling patients to actively participate in self-care^1^. Children with T1DM and other chronic diseases need to comply with a broad range of recommendations that demand new skills, knowledge, and behavioral changes^1^ ^,^ ^4^, particularly towards self-care tasks. Thus, the alliance to achieve disease self-management depends on strategies and techniques based on behavioral theories taking into account the health care team, family’s support, and the child biological/psychosocial development^5^ ^-^ ^7^ and preferences^1^ ^,^ ^8^. 

Interactive technologies such as video games have proven to be powerful intervention tools to achieve positive effects on behavioral change, and consequently, promote adequate disease self-management^9^ ^-^ ^13^. Nevertheless, the literature points to the need to strengthen the use of behavioral theories and its behavioral determinants^14^ and how to apply theoretical frameworks in video game designing to achieve health-related behavioral changes^8^ ^,^ ^10^ ^-^ ^11^ ^,^ ^14^ ^-^ ^15^.

In addition, studies have emphasized the importance of the participation of future users in all phases of development, that is, from conception to final evaluation, in the development of effective games^8^ ^,^ ^11^ ^,^ ^16^ ^-^ ^17^. The User-Centered Design (UCD) is highlighted among studies that address the development of interactive health technologies, which incorporates user participation in the several phases of the development process^8^ ^,^ ^11^ ^,^ ^16^ ^,^ ^18^ ^-^ ^21^. In addition, the literature^17^ points the role of the qualitative research to video game development, which is an important step that needs to be followed by studies that are designed for teaching-learning and behavior changes.

Studies have reported considerable improvements that enhance the effectiveness of video games and further explore their potentialities. Beyond the user´s involvement in the designing process^8^ ^,^ ^16^ ^,^ ^19^, the use of theories and theoretical frameworks (TF) are important keys in designing video games and interactive technologies that can induce behavioral changes^11^ ^,^ ^22^ ^-^ ^24^. 

Some video game interventions designed for children with T1DM^9^ ^,^ ^25^ ^-^ ^26^ and health in general^27^ are described in the literature. Theories as The Elaboration Likelihood, Social Cognitive Theory (SCT), Self-Determination Theory (SDT), Behavioral Inoculation, and Transportation Theory are examples of health behavioral theories considered in studies that structured frameworks designed to prevent type 2 diabetes mellitus (T2DM)^23^ ^,^ ^15^ and to influence changes in diet and physical activity^22^ ^,^ ^28^ ^-^ ^29^. The Theory of Planned Behavior guides a framework designed to increase adherence to treatment in T1DM patients^26^. The Theory of Multiple Intelligences, the SCT, and the game elements form a model to guide researchers to create games for learning and behavioral changes^27^. All these researchers have played an essential role in the advances to achieve well-designed and well-implemented video game interventions.

Among the health behavior theories, the Transtheoretical Model of Change (TTM), originally used as a conceptual tool for smoking cessation^30^, has been used in a growing range of investigations. The promotion of physical activity among adults with T1DM and T2DM^31^, other behavioral changes in diabetes^31^ ^-^ ^34^, increased consumption of fruits and vegetable among adolescents^35^, and promotion of effective stress management^36^ are discussed. According to the TTM, individuals with the same conditions may present five different stages of change: pre-contemplation, contemplation, preparation, action, and maintenance. It predicts that applications need to cater to different individuals and different moments in their treatment^37^. Although the use of this theory is part of recent literature discussions, no video game design has considered it^38^. 

Although the literature presents many studies that propose the development and Evaluation of video games for health, the scientific community warns about the importance of rigorous development of these educational technologies. The studies must follow theoretical foundations and also consider the needs and experiences of the future user^8^ ^,^ ^17^ ^,^ ^21^.

In order to contribute to this field of studies, we conducted a methodological study that address the following research questions: 1. What is the conceptual framework to guide the designing of video games for children with T1DM that is based on the TTM, behavioral determinants from other health behavior change theories, and the UCD approach? 2. What are the designing principles for a video game for children with T1DM that are based on a theoretically based conceptual framework? The motivation behind this methodological research study focuses on presenting a theoretically based conceptual framework for designing video games for children with T1DM. We expect with this study to contribute with researchers in the technologies development field to improve methodological issues important to the design of the video games, as well as to contribute with those who aim to develop technologies for children with chronic diseases, especially with T1DM.

## Methods

**Figure 1:**
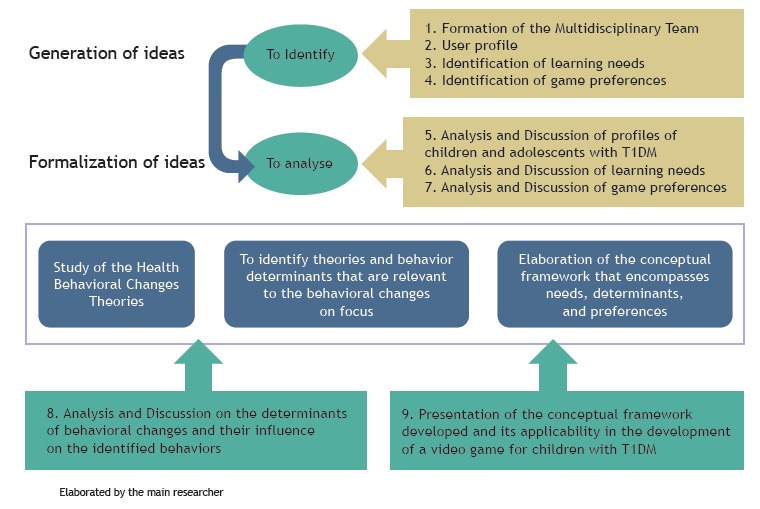
Representation of steps completed for the development of the conceptual framework that will support the development of a video game for children with type 1 diabetes mellitus. Ribeirao Preto, SP, Brazil, 2013-2015.

This is a methodological study that describes the steps for the development of the conceptual framework in view of the health behavioral change theories and the UCD approach as theoretical and methodological frameworks, respectively. Figure 1 shows steps 1-9 completed for the development of the conceptual framework.

The generation and formalization ideas consisted of steps one to seven. A multidisciplinary team, formed by seven professionals, contributed in the entire process. Children were recruited at the Endocrinology and Childhood Diabetes Outpatient Clinic from the General Hospital of the Medical School of Ribeirao Preto at the University of Sao Paulo, Brazil. A survey on the profile of children and adolescents who are future video game’s users was carried out. Subsequently, six focus groups (FG) were conducted with the participation of 19 children (5 boys and 14 girls), aged 7 to 12 years. The learning needs regarding knowledge about the disease and self-care tasks were identified and analyzed^39^. In a second round, five FG were conducted with the participation of 15 children (4 boys and 11 girls), aged 7 to 12 years, to confirm and discuss the ideas generated and the clientele’s preferences regarding the design of a video game that represented them. The double participations resulted from the number of follow-up consultations the children had at the outpatient clinic and their willingness to participate, not configuring restrictions or exclusions from the FG. All the process of this formative research counted on 21 children and 39 participations, from December 2012 to August 2013. The eligibility criteria included children (boys and girls) aged 7 to 12 years with a diagnosis of T1DM, regardless of the time of diagnosis. The exclusion criterion was any form of developmental delay that could interfere with the data collection strategy. The focus group data were analysed following deductive and inductive content analysis guidelines^40^ ^-^ ^41^.

Step eight concerns the in-depth study of several health behavioral change theories and their determinants, culminating with the choice of appropriate theories and determinants that should be capable of influencing children’s behaviors and preferences identified. Step nine presents the conceptual framework that guides the development of video games for children with T1DM, completed in November 2015. The study was approved by the Institutional Review Board (Process no 246.418).

## Results

The TTM was chosen as the theory that will form the basis of the developed conceptual framework. To it, we incorporated behavior determinants along the stages of change according to the needs identified in the formative research. The research based on assumptions of the TSC(42-43), Theory of Self-Determination(44), TTM(45), and the Elaboration Probability Model(46). The determinants of self-efficacy, knowledge, self-regulatory skills, outcomes expectations, intrinsic and extrinsic motivation, goal setting, and social support are applied throughout the conceptual framework. These were applied to each stage of change proposed by the TTM and are aligned to a specific composition in the created game plot according to the needs and game preferences identified.

**Figure 2:**
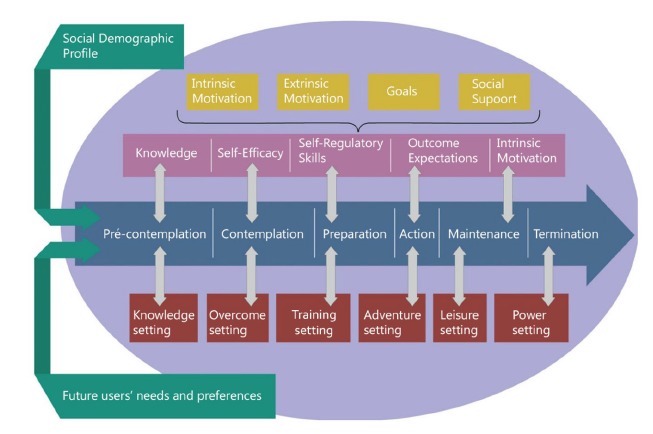
Conceptual framework: foundation to guide the development of video games for children with Type 1 Diabetes. Ribeirao Preto, Sao Paulo, Brazil, 2015.

Figure 2 illustrates the conceptual framework created. Stages of change appear in blue; the behavior determinants in each stage of change appear in pink. The intrinsic and extrinsic motivation, goals, and social support, applied to all stages, are displayed in yellow. Each of the stages of change constitutes a level in the proposed game, where the player, after fulfilling the tasks and scheduled interventions, will be able to move to the next level. The scenario in which each phase of the game will happen is represented in red. The analysis of the appropriate health behavior determinants and their relation with the children preferences and ideas about the game in each stage of change allowed authors to the discovery of these terms. The chose for each game’s scenario will be presented in the results’ section. Our model is structured within a circle^47^ that will promote to the perception that the child is gaming and playing in a space that is not like “ordinary” life. The profile of the future user, and his learning needs and preferences previously identified are outside circle in green. The interventions proposed by the conceptual framework for each phase will be presented below. The testimonials from children participating in the study illustrate the results.

In the pre-contemplation stage proposed by the conceptual framework, the child has no information about diabetes or does not believe in the importance of proper self-care for disease management. The determinant of knowledge, from the TSC, is the key to moving the child from pre-contemplation to the stage of contemplation^48^. In addition, the learning needs about illness and self-care, evidenced in the children’s voices, justified the implementation of this determinant: *In diabetes, there is no insulin to kill sugar (...) because if it did not kill sugar, diabetes would always be high* (Girl, 8).

We named “The discovery setting” the place that can simulate the location of diagnosis, outpatient follow-ups, or emergencies resulting from treatment failures. In this stage, the child does not consider a change in the near future, so he is not willing to talk or reflect on his current behavior. A health team prepared for the use of creative and interactive education strategies, other children with diabetes and family represent the determinant of social support determinant. Several tasks can promote gradual learning through situations in which children have to make choices that will immediately reflect on the child’s health condition in the game. In this stage, the video game will promote an immediate feedback on the player’s decisions, functioning in an action-reaction scenario, alerting the player whether he is on the right track or not. Experienced risks and benefits will increase their knowledge. The following testimonial illustrates on how the game could be developed to increase the knowledge about the self-care: *The character will have to enter the body to try to rescue the insulin. If he applies in the spots with lumps in the skin* [referring to lipodystrophies]*, the insulin will get stuck in the skin and the player will have to rescue it* (Girl, 10).

The extrinsic motivation, a determinant from the SDT, will be in high levels in this stage, because the child at this moment does not think about modifying his behavior and needs to be stimulated. Rewards, feedbacks with informational characteristics, and positive comments coming from professionals, friends, and parents will be key components to motivate the child.

We believe that these strategies can motivate the child to move to the next stage of behavioral change. In the stage of contemplation, the child must overcome barriers that will allow a change in the coming months. Pain, fear, insecurity, desire, and anger were identified from the perspective of children as barriers to adequate self-care tasks. A 10 years old boy exemplifies how the video game should be at the time of insulin injections to help him to overcome the fear: *To pass this level, the character has to get the insulin that will be inside a refrigerator. But there will be a “monster”. The character will have to kill it in order to get the insulin* (Boy, 10).

Self-efficacy from the TSC will be paramount at this stage. The video game should include tasks that help the child to overcome barriers to treatment, or even make them easier, which is in line with the goals set for this phase. “The overcoming setting” can represent the child’s home, school, or restaurants. Self-instruction and dissociation strategies may increase feelings of self-efficacy in terms of pain felt by the child during finger punctures, for example. Distraction techniques or the use of therapeutic toys may direct the child to something more pleasurable. The child can also be invited to imagine pleasant moments and images. The support and guidance from friends, health professionals, and family members constitutes the social support applied to this stage.

The self-care tasks will be simplified in order to have the player practicing them in stages, promoting confidence. The testimony of a 12-year-old girl illustrates how the game could approach the issue of diet: *For each food that the player chooses, a question about diabetes is prompted. If he wants to eat a food, he clicks the food image. A new screen opens in the game with a question that the player will have to answer choosing an alternative answer* (Girl, 12).

Extrinsic motivation will be applied in order motivate the child to perform tasks that do not bring pleasure such as insulin injection or self-monitoring of blood glucose (SBMG). The player, in the role of the child, receives incentives, rewards, or points for each moment in which he is more confident about these barriers. The challenges faced in this stage, the self-perception of overcoming, and the social support during the accomplishment of the task and after its conquest, will be promoters of intrinsic motivation in disease management.

When in the preparation stage, the child already thinks about changing in the near future and has already taken some steps toward this action. So, the child will dedicate himself to the completion of a plan aiming at the desired change; he can soon experience small transformations as his determination to change is increased. The player will perform self-care tasks and experience a progressive improvement in his practical skills. These changes may be noticed from the moment he decides to include fruits and vegetables in one of his meals, choose different sites of insulin injection, or even learn to perform the carbohydrate count. A 12-year-old girl suggested a task: *The player had to click on that little strip, on that little black marking* [referring to the plunger in the syringe]*. In the game, we had to click and go on clicking (...)* [referring to having to click until filling the syringe with the medicine in the desired quantity] (Girl, 12).

We name this location as “the training setting”. This phase can take place in a diabetes camping, an educational session or weekend meeting. Friends and health professionals will form the social support necessary to assist the child in learning and strengthening the practical skills required for meals and snacks, in the SBMG, insulin injections, and various recreational activities. The determinant of self-regulation skills, from the TSC, is applied. The main character will solve problems related to his difficulties in self-care tasks. Goals will be established with the opportunity of self-evaluation regarding his progressions and achievements. 

The levels of intrinsic and extrinsic motivation will be matched. At this point, the child will continue to receive external incentives such as points, prizes, or tokens to exchange, during the execution of his activities. However, he will be free to choose tasks and activities. The setting will stimulate his autonomy and sense of competence, step by step, which will be reflected in the care of diabetes. The player will meet other children, which will help him to feel like a member of a group connecting and relying on it. The activities in this stage aim at overcoming the difficulties in practical skills and encouraging the child to apply the expertise and knowledge gained in moving to the stage of action.

In the stage of action, the child will put into practice the changes that have been achieved, which will continue to increase. It comprises the fourth phase of the framework and represents “The adventure setting” considering that this is the moment of the child’s action. The content seized by the child so far will be contextualized in a way that is meaningful to him. An adventure with friends, experiencing unprecedented situations, will involve the child and lead him to perform the required actions in the role of the main character. A child suggested a game setting: *We wanted to go to new places. A park, a zoo, a woodland* (Girl, 10).

The levels of extrinsic motivation will be reduced, as opposed to those of intrinsic motivation. The stimulation for self-direction, initiated in the preparation stage, will be intensified and the child will experience situations in which decision making will be essential for his progression in the game. The player’s choices will result in positive or negative consequences, and in an appropriate and informational way, the game will bring him feedback. Friends will provide the necessary social support in the face of proposed challenges, which aim to encourage the child’s decision-making regarding actions related to diabetes self-care. 

However, the children’s testimonies showed a lack of awareness of the consequences of their actions: *When I guess the result of blood glucose testing will be high, I do not take the test* (Girl, 12). In order to influence positive behaviors in this stage of action, our focus will be on making the child believe that by behaving in a certain way he will have this behavior reversed into good results for his treatment. This will influence the child to maintain certain actions, hoping that similar results may occur again, which characterizes the influence of the determinant of expectations through results, from the TSC. 

The child will experience physical results from his actions such as how to recover quickly when performing the appropriate treatment for hypoglycemia. The child can also perform appropriate actions based on expectations of appreciation from people who are significant to him such as those in the health staff and family. Finally, the well-being caused by performing appropriate behaviors will determine positive self-assessments, which will influence the child to maintain such behaviors. These strategies and experiences will help change the child’s behavior by moving him to the next stage of change called maintenance.

In the maintenance stage, the child will retain the behavioral change achieved so far, preventing relapses and showing more confidence that the changes may continue. “The leisure setting” was developed to attend to the preferences of the children. Leisure moments in barbecues, birthday parties, and amusement or water parks are those where maintaining proper behavior is extremely demanding and difficult. At these situations, the child should demonstrate knowledge about the disease and self-care tasks, be confident, present adequate practical skills for self-care tasks, and act in a way that achieves good results. An 11-year-old boy illustrates the children’s preferences. *He* [the player] *is in a barbecue. Before eating, he has to do the carbohydrate count, inject insulin correctly, and then eat, drink soda, and other things* (Boy, 11).

In this stage, the levels of extrinsic motivation, that is, of external stimulation, will be low in relation to the high levels of intrinsic motivation. Intrinsic motivation, applied gradually in previous stages, will reach high levels and will be the main determinant in this stage of change, motivating the child to maintain the behaviors achieved and to continue his progression. The focus is on preventing relapses into inappropriate behaviors that were present in earlier stages. The child, in the role of the main player, will be encouraged to expand and exercise his own abilities, exploring and learning in a challenging environment in order to maintain feelings of competence, autonomy, and connectivity^44^. The idea is to make the game more rewarding to the child as he feels more capable of performing self-management without offers of outside rewards.

Parents, friends and teachers will be the social support necessary for the child to feel part of a group. Children can receive help from friends during play time; family members can encourage the child and teachers can show support during a school field trip. The game should be structured in a way to provide feedback to the child as the result of his choices, which may come from people who are relevant to the child. Positive feedback and adequate communication, even in situations that do not represent adequate disease management can provide feelings of competence. The opportunity of free choices in these environments will foster a sense of autonomy.

The child will feel more confident to experience moments of leisure without the difficulties that could be imposed by self-care tasks, which could happen to a child in the stage of contemplation, for example. At the end of this stage, we expect the child to be intrinsically motivated, that is, he will value self-care in diabetes and show a sense of personal commitment to his treatment, overcoming the lack of knowledge, barriers, difficulties in practical skills, and the lack of awareness, maintaining and evolving every day as the result of achieved behavioral changes.

In “the power setting”, the main character in the role of a superhero will be expert and knowledgeable in T1DM. He will demonstrate knowledge about the disease and self-care tasks. Confident, he believes in the positive outcome of his actions and is the champion of T1DM management, proving to be intrinsically motivated to do so. Being attractive and displaying the high power of persuasion^46^, this superhero will be able to transmit his knowledge and abilities to other children with diabetes who will be in the previous stages of behavioral change.

In this stage, the superhero and other children with T1DM will face difficulties, and together will carry out various activities to overcome them. Through their behaviors and convincing messages, the superhero, envisioned as a role model, will encourage these children to try and embrace positive changes. The goal is to promote the perception that the child can overcome all his difficulties towards T1DM self-management and can still act as a model to be followed by other children who already have similar experiences.

## Discussion

The development of this conceptual framework solidifies the whole process of development of this research that used the health behavioral change theories and its determinants as a theoretical reference, as well as the UCD as the methodological referential.

The framework presented is inside a circle. According to the literature, the spatial, temporal, and psychological boundaries that exist between the game and the real world are what characterize the “magic circle”^47^: the space where the game happens. The child’s real world has a special meaning when placed inside the “magic circle”, which can promote significant experiences to the player^47^ ^,^ ^49^. 

The TTM is the theory that is the base of the conceptual framework. It recognizes the individual stages that are required to develop a behavior. It suggests that the individual moves through stages when changing existing behaviors or adopting new ones^45^. Considering the stages of change, the literature recommends a combination of interventions and information, personalized to each stage, which facilitates individual behavioral changes^45^ ^,^ ^48^ ^,^ ^50^. Also, the TTM theory allows that different behavioral determinants from other theoretical models (TSC^42^ ^-^ ^43^, Theory of Self-Determination^44^) are applied in interventions across the stages of change, which justifies its name as transtheoretical theory^45^, as we proposed in the conceptual framework. In this study, we choose the health determinants that should be capable of influencing children’s behaviors and preferences previously identified, which is a differential in studies from this field.

Taking into account a chronic disease such as T1DM, the understanding of the disease and achievement of self-management are particular stages in each individual^51^. A study that identified learning patterns in patients older than 18 years who were recently diagnosed with diabetes, concluded that even within the same disease duration, the learning needs, strategies, and health care requirements for living with diabetes are different between individuals^51^.The understanding and self-management of diabetes occur gradually and according to the stage of change in each individual^51^. The TTM places patients in their most appropriate stage of change through an individualized assessment of readiness. The complexity of diabetes self-management such as the acquisition of knowledge, skills, and behaviors necessary for appropriate self-care tasks such as insulin injections, self-monitoring blood glucose testing (SMBG), diet, and exercises can be approached by the TTM^48^ and is considered in the video game design for children with T1DM at this conceptual framework.

 The video game proposed by the application of the TTM identifies key behaviors related to the disease and plans and strategies in each stage, helping the patient to move through the stages of change, enhancing and facilitating self-management in diabetes^48^. It is important to remember that a child can be at one stage in the dietary behavior and in another in the insulin injections^48^. In applying this theory, the videogame can work with the tasks of self-care, placing them in different stages for the same individual as well as with other aspects necessary for disease self-management. The conceptual framework that is presented in this study can assist health professionals and designers to develop a particular strategy that will benefit a greater number of individuals. 

According the literature, the pre-contemplation stage is aimed to those who have attempted to change behaviors and failed, or were unable to change or maintain change^38^ ^,^ ^45^ ^,^ ^48^. In this stage the individual is not prepared to overcome barriers or implement care practices^38^.The videogame needs to surprise and convince the child that change is possible^38^ using stories that are easily entertaining and funny. The literature points that the immediate feedback on the player’s decisions, functioning in an action-reaction scenario, alerting the player whether he is on the right track or not^22^ ^,^ ^25^ are successful strategies to promote learning about the disease. Medals, scoreboards, points, challenges, tests, feedbacks with informational characteristics, and positive comments^52^ will also be key components of the game to motivate the child. Also, family, health team and friends will represent the determinant of social support^27^ ^,^ ^43^ ^,^ ^53^, essential to this stage. Thus, the child will have reasons to positively modify his behaviors in relation to treatment^42^.

In the contemplation stage, the self-efficacy is a determinant capable of influencing the child’s belief that it is possible to overcome obstacles by adopting new habits^42^. By believing that he can prevent or face moments of fear or pain, the child raises their levels of self-efficacy, having no more reason to feel disturbed by these moments^42^. The video games can include tasks that help the player to overcome barriers to treatment, or even make them easier^54^. To carry out the insulin injection in a plush toy, and thus losing the fear of handling syringes and needles, is one example^55^. Older children may respond better to cognitive behavioral therapies that include relaxation, coaching, guided imagery, behavioral testing, and reinforcement^55^. Relaxation techniques can be organized by a meaningful person and promote relief of pain, affliction, anxiety, and stress^56^ ^-^ ^59^ leading the child to feel more confident that he is able to perform certain activities^42^.

When feeling confident about executing short-term goals, the child overcomes insecurities, feels motivated, and self-efficacy is increased until the entire task is carried out^42^ ^-^ ^43^. A well-developed narrative that reaches the child’s engagement in the activity can lead to potent and positive stimulation^59^. When the stimulus is interpreted positively, the belief in individual efficacy is increased^59^ and the child will feel able to overcome barriers, and subsequently move to the next stage of change proposed in the framework.

In the preparation stage, the child will dedicate himself to the completion of a plan aiming at the desired change; he can soon experience small transformations as his determination to change is increased^54^. The diabetes camping’s have represented effective strategies in the promotion of adequate diabetes management^60^ ^-^ ^63^ with the aim of sharing experiences with colleagues with the same diagnosis and to empower children to become more responsible for their condition. Thus, the scenario of diabetes camps are ideal places for teaching self-management skills^60^ as well as weekend meetings and innovative and creative educational group sessions. 

According to the literature, the use of stories can motivate the child to continue playing the game, encouraging him to engage in healthy behaviors, even without the presence of extrinsic motivation^64^. In the action stage of the framework, the intrinsic motivation determinant will increase the feelings of autonomy, connectivity, and competence gradually increasing the child’s intrinsic motivation in the management of diabetes^44^. The child will retain the behavioral change achieved so far, preventing relapses and showing more confidence that the changes may continue^45^ ^,^ ^65^. At this point, the child will be at the maintenance stage of the framework, which will prepare the player to his progression to the last stage.

Most studies do not include the termination stage of TTM in the interventions because in this stage, individuals are 100% self-efficacious, which is a difficult condition to achieve^45^. In this stage, there is no chance for re-engaging in inappropriate behaviors. Even in difficult moments, the individual is totally confident and sure that he will not return to previous behaviors. However, we will use this stage in the conceptual framework in which the child, in the role of the main character, will be considered a superhero and will be able to help other children who are in the earlier stages of change.

The Elaboration Probability Model^46^ used in the last stage refers to results that different persuasion variables have on the change process and in the strength of these results^46^. Superheroes have a high impact power in the real life of children, who can idealize their growth, thinking of being like the hero they most admire^66^. Comic superheroes have played important roles promoting health in various fields. Their interventions in the area of epidemiology, education, and psychology have been reported^66^. The use of heroic characters in the history of video games, aiming at promoting health for children, is a strategy that has been used^23^ ^,^ ^67^.

From the analysis and integration of the steps developed in this research, the conceptual structure was presented. Future research may examine the learning needs of target populations in other age groups such as adolescents or even considering other chronicities such as cancer, asthma, and cystic fibrosis. Also, the children who participated in the FG were recruited from a diabetes education group, which is considered as a study limitation. It may be that, if we had included children who were not participating in educational groups, different learning needs could be identified.

## Conclusion

The presented conceptual framework places the child with T1DM in the different stages of behavioral change, based on the TTM, applied behavioral determinants from other theories and involved the future users using a UCD approach.

We chose support on the broad scope of this conceptual framework because we know that a child may be in the pre-contemplation stage regarding insulin injections and in the action stage regarding the SBMG. When playing a video game that contains all the stages presented in this conceptual framework, the child will receive interventions for his needs at different levels, which confers the probability of reaching a greater number of children in different situations.

In addition, the conceptual framework is developed in a way that different games can be structured and developed, depending on the identification of the needs of future users and the stage of change in which they are. It is imperative to remember that the profile of the target population as well as its needs and preferences must be identified in advance. The involvement of the target population in this study and identification of its needs and preferences, allowed us to identify theories and determinants capable of promoting positive changes in health behaviors, and to survey and include video game elements in the applicability of the conceptual framework developed. We are confident that it contributes to the development of future research whose objective is the development of video games for children with T1DM and other chronic diseases. The creation of these educational strategies, using and testing this model, and based on learning needs and preferences identified in its own users, can contribute to the advancement of knowledge in this area.
